# A Fast and Reliable Method to Interpret Short-Term Mortality in Perforated Peptic Ulcer: Red Cell Distribution Width is Sensitive and Specific

**DOI:** 10.1155/2021/5542619

**Published:** 2021-05-18

**Authors:** Okan Murat Akturk, Mikail Çakır, Yaşar Murat Vardar, Doğan Yıldırım, Muzaffer Akıncı

**Affiliations:** Department of Surgery, University of Health Sciences, Haseki Training and Research Hospital, Istanbul, Turkey

## Abstract

**Introduction:**

Peptic ulcer is an important health problem worldwide with a prevalence of around 5%. Peptic ulcer perforation is a potentially mortal complication of peptic ulcer disease. We aimed to investigate the potential use of red cell distribution width as a prognostic marker in peptic ulcer perforation.

**Methods:**

The files, operation notes, biochemical and hematological parameters, and prognosis of patients who were operated for a peptic ulcer perforation were reviewed in a retrospective cohort study. The relation of red cell distribution width (RDW) to main outcome in-hospital mortality was assessed.

**Results:**

The mean age of the 172 patients was 40 ± 17.89. There were 158 (92%) males and 14 (8%) females. The in-hospital mortality was 8.7% (15/172). The median RDW in the group with mortality was 15.00 (interquartile range (IQR): 14.30–17.20) compared with the median RDW in the group with no mortality as 13.2 (IQR: 12.80–14.00, *p* ≤ 0.001). Receiver operator characteristic curves were plotted for RDW to identify nonsurvivors and yielded a significant area under the curve as 0.812 (95% confidence interval: 0.682–0.942). The sensitivity and specificity of RDW at a cutoff value of 14.25% were calculated with an accuracy of 81.98 (95% confidence interval: 75.40–87.41) as 80.00 (51.91–95.67) and 82.17 (75.27–87.81), respectively.

**Conclusion:**

Increased RDW may be of use to interpret mortality in patients with peptic ulcer perforation.

## 1. Introduction

Peptic ulcer perforation (PUP) is a serious complication of peptic ulcer disease (PUD) that causes morbidity and mortality. The lifetime prevalence of perforation in patients with PUD is reported to be around 5% [1]. Despite advances in the treatment of PUD, perforation occurs in 2–10% of patients and accounts for more than 70% of deaths associated with the disease [2]. The onset of PUP is significant, rendering diagnosis thereof relatively easy and allowing for rapid intervention. Mortality rates are reported to be between 1.3% and 20% [3]. Due to its relatively common occurrence, identification of patients at a greater risk of mortality at earlier stages would result in better allocation of resources regarding intensive care and transportation and earlier medical intervention.

Red cell distribution width (RDW) reflects heterogeneity among erythrocyte volumes, and elevated RDW levels indicate an increase in red blood cell size variation called anisocytosis [4]. RDW is easily obtained during routine complete blood count (CBC) lab testing, and accumulating evidence demonstrates that RDW is a valuable prognostic tool in multiple disease settings.

RDW, as a novel inflammatory marker, is suggested to be associated with type 2 diabetes mellitus, coronary artery disease, malignant conditions, lumbar discopathies, respiratory distress syndrome, and multiple myeloma [5–10]. An elevated RDW has been associated with poorer outcomes including increased mortality in patients with ischemic heart disease, acute mesenteric ischemia, cerebrovascular disease, and renal cancer [11–14].

RDW is reflective of inflammation, and in the general population, a higher RDW is associated with increases in ESR and the inflammatory markers IL-6, C-reactive protein, and receptors for TNF I and II [15]. The exact mechanism underlying the link between clinical manifestations of elevated RDW and the diseases mentioned above has yet to be fully elucidated, but high levels of RDW have been associated with inflammation, poor nutritional status, and changes in erythropoiesis [16, 17]. This study investigates whether RDW can predict the prognosis of PUP patients upon hospital admission.

## 2. Materials and Methods

We evaluated the in-hospital mortality of patients receiving surgery for PUP at the General Surgery Department of Istanbul Haseki Training and Research Hospital in a retrospective cohort study. The medical records of patients who underwent emergency surgery for PUP between May 2013 and May 2017 were reviewed according to codes designated by the International Statistical Classification of Diseases and Related Health Problems, 11^th^ revision. This study was approved by the local ethical committee (195/26.07.2018) and conformed to the principles of the Declaration of Helsinki. Written consent to use their clinical data was obtained from patients before surgery. Preoperative diagnoses were based on clinical data and radiological findings, and chest radiogram was the most common diagnostic tool. Nearly half of the patients underwent computed abdominal tomography (CT) for differential diagnosis unless contraindicated. In addition, biochemical tests on hepatic and renal function and hemograms are routinely obtained from patients planning to undergo surgery. RDW was routinely measured as part of an automated CBC count using a hematology analyzer. The reference range of RDW at our hospital is 11.5–14.5%. All operations were carried out under general anesthesia, either via midline incision or laparoscopy, and all patients underwent resuscitation with individualized fluid-electrolyte support therapy. Patients younger than 16 years of age or with a hematologic disorder, recent transfusion history, or recurrent perforation were excluded from the study. The primary outcome analyzed was in-hospital mortality rate.

## 3. Statistical Analysis

The main goal of this study was the prediction of mortality in the patient group. All statistics were performed using SPSS 22.0 for Windows (SPSS Inc, Chicago, IL, USA). Data were checked for normality with the Kolmogorov–Smirnov test and found to be nonnormally distributed and are thereby expressed as median and interquartile range (IQR). Categorical variables were expressed as frequencies and percentages. Patient age and sex, American Society of Anesthesiologists (ASA) physical status score, diameter of perforation area (mm), and the following laboratory parameters were analyzed: white blood cell count (WBC; 10^3^/mm^3^), RDW (%), serum albumin (g/dL), creatinine (mg/dL), total protein (g/dL), blood urea nitrogen (BUN; mg/dL), and potassium (mmol/L). A binary logistic regression was carried out to reveal variables that were significantly associated with mortality. Fischer's exact test was used for intergroup comparisons, the Mann–Whitney *U* test was used for nonnormally distributed continuous parameters, and Pearson's chi square test was used to analyze categorical variables. All tests were two sided, and a *p* value of less than 0.05 was considered statistically significant. An analysis of receiver operating characteristics (ROC) curve associated with the area under the curve (AUC) was used to derive optimal cutoff values and their specificity and sensitivity to predict progression to mortality. The AUC also indicated the probability of concordance between the predicted probability of postoperative mortality and the actual postoperative state. Determinant factors were analyzed using logistic regression analysis.

## 4. Results

**Figure 1:**
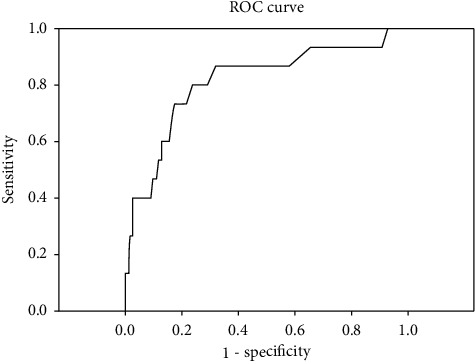
Receiver operating characteristic (ROC) curve analysis for RDW to identify nonsurvivors.Area under the curve (AUC) is 0.812 (95% CI: 0.682–0.942).

**Figure 2:**
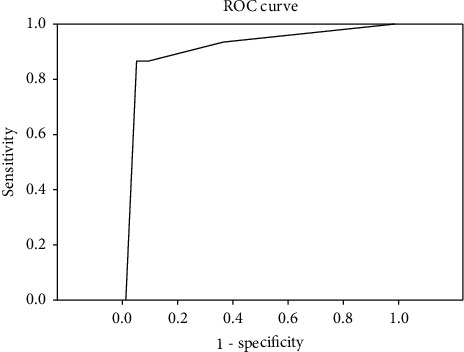
Receiver operating characteristic (ROC) curve analysis for ASA physical scores.Receiver operating characteristic (ROC) curve analysis for ASA physical scores revealed a significant AUC of 9.11 (95% CI: 0.87–1.00).

The median age of the 172 patients was 40 ± 17.89 years (range 18–93). There were 158 males (92%) and 14 (8%) females. Patient characteristics are provided in Table 1. All patients underwent surgery as a primary treatment. Of these, 140 (81.0%) patients underwent open repair with omental patch, 17 (9.7%) received laparoscopic repair with omental patch, 9 (6.0%) underwent gastrojejunostomy, 2 (1.1%) received gastrectomy with truncal vagotomy, 2 (1.1%) had a Roux-en-Y (RNY) gastrojejunostomy, and 2 (1.1%) had a tube duodenostomy. In two cases, appendectomy was added, as well. Fifteen patients (8.7%) died during the 30 days following the operation. Overall median RDW was 13.3 (IQR: 12.80–14.30). The median RDW in the mortality group was 15.00 (IQR: 14.30–17.20), whereas the median RDW in the survival group was 13.20 (IQR: 12.80–14.00; *p* ≤ 0.001). ROC curves were plotted for RDW values to identify nonsurvivors with a significant AUC (0.812; 95% CI: 0.682–0.942; Figure 1). The sensitivity and specificity, positive likelihood ratio (+LR), and negative likelihood ratio (−LR) for RDW at a cutoff value of 14.25 were calculated to an accuracy of 81.98 (95% CI: 75.40–87.41; Table 2). ROC curve analysis for ASA physical scores revealed a significant AUC of 9.11 (95% CI: 0.87–1.00; Figure 2).

In binary regression analysis, the variables age (*p* ≤ 0.001), ASA score (*p* ≤ 0.001), WBC (*p* ≤ 0.001), albumin (*p* ≤ 0.001), total protein (*p* ≤ 0.001), diameter of perforation (*p*=0.01), BUN (*p* ≤ 0.001), and potassium (*p* ≤ 0.001) were found to be significantly associated with mortality (*p* ≤ 0.001), but creatine was not (*p*=0.17) (Bonferroni correction).

## 5. Discussion

PUP is a significant global health problem, constituting a surgical emergency that is potentially mortal [18]. While the prevalence of PUD has decreased globally in recent decades, this reduction has not been accompanied by a decrease in complications arising from peptic ulcers [19]. Rates of mortality following a PUP have been reported to range between 8.5% and 25.3%. Thomsen et al. [20] in a retrospective cohort of 2,061 patients reported a mortality rate of 25.3%; Noguiera et al. [21] reported a mortality rate of 10% in 210 patients; and two Turkish studies reported mortality of 8.8% and 8.6% [22, 23]. In our study, the in-hospital mortality rate was found to be 8.7%, which closely aligns with results from the Turkish studies.

There are several classification systems for predicting severity of disease in PUD patients. The Boey score was the first to be developed and remains popular for predicting mortality in PUP using three major variables: major medical illness, preoperative shock, and a perforation duration greater than 24 hours [24]. Boey score was able to correctly predict mortality in 93.8% of PUP patients in a prospective study [25].

Another system for prediction of short-term mortality is the peptic ulcer perforation (PULP) score, a method that evolved from a multicenter study on 2,668 patients in Denmark. The PULP comprises 8 variables associated with poor prognosis: age over 65, active malignant disease or acquired immunodeficiency syndrome, liver cirrhosis, steroid use, length of time elapsed between perforation and admission, preoperative shock, raised serum creatinine level, and an ASA grade over I [26]. The AUC for the PULP and Boey scores was also calculated, with results of 0.83 and 0.70, respectively.

While the ASA assessment provides a general score for surgery suitability, it is also a commonly used risk score for PUP. In our study, the AUC for ASA was 0.912, similar to results from a 152 patient study by Lohsiriwat et al. [27], which reported a mortality rate of 9% and an AUC of 0.91. In another study of 117 patients, Buck et al. [28] reported an AUC for ASA in patients with PUP of 0.73 and a mortality rate of 17%. Moller et al. [29] studied ASA scores in a group of 708 patients and found an AUC of 0.78 with a mortality rate of 27%. ASA is an assessment of overall patient health based on five signifiers, and mortality risk increases with higher values [30]. However, the ASA score has been reported to have interobserver variability [31].

PULP combines properties introduced by the Boey score and PULP, scoring slightly better than both systems in the prediction of mortality [29]. The Boey score was developed using American patients in the early 1980s, while PULP is a more recently derived classification system (Table 3).

Significant risk factors which may cause death are the presence of shock at admission, comorbidities, resection surgery, female, elderly patients, a delay in presentation of more than 24 h, metabolic acidosis, acute renal failure, hypoalbuminemia, being underweight, and smokers [3].

RDW was found to be strongly associated with all causes of mortality in both middle-aged and elderly adults in a large cohort study, and mortality rates were particularly elevated in patients with an RDW greater than 13.4; for every 1% increase in RDW, mortality risk increased by 22% [32]. The underlying mechanisms associated with RDW elevation are as yet not fully understood. Erythropoietin in serum increases along with aging to compensate for subclinical blood loss, increased red blood cell turnover, or increased erythropoietin resistance in red cell precursors [33]. This is thought to lead to an overall higher RDW in older patients. Erythropoietin itself might also be partially responsible for changes in red cell volume and contribute to the predictive qualities of RDW.

RDW has been shown to be associated with mortality in general, in addition to risk of death from cardiovascular disease, cancer, and chronic lower respiratory tract disease, even after adjusting for anemia and related nutritional deficiencies in outpatients [34]. Lippi et al. [35] reported a graded association of RDW with high-sensitivity C-reactive protein and erythrocyte sedimentation rate, independent of several confounding factors.

Given that an elevated RDW is associated with comorbidity and inflammatory status, it alone might substitute for scoring systems that incorporate patient fitness, such as ASA score or inflammation. Moreover, the time lapse between the onset of symptoms and hospital admission may lead to higher inflammatory status in cases of delayed intervention.

In this study, we suggest that RDW is a specific and sensitive laboratory marker that can be used to predict mortality in patients with PUP. As far as we know, this is the first study evaluating RDW for prediction of PUP patient mortality.

There are certain limitations to our study, the first of which being that it is a retrospective study. Also, as it is not a multicenter study, selection bias is inevitable. Our study population may be younger than PUP sufferers in general, as well, because our hospital serves a large population of foreign-born residents who may have lacked adequate previous medical care. Our study reflects a low positive predictivity, although a very sensitive one, potentially because of the relatively low prevalence of the studied variable: mortality. Besides, it is not proposed as a substitute for available risk scoring systems but provides clear foresight for the surgeon; thus, this field needs additional studies.

## 6. Conclusion

Increased RDW in preoperative assessment may be a sensitive and specific indicator of mortality likelihood in patients with PUP. Surgeons can employ elevated RDW as a prognostic factor and should carefully monitor patients with increased RDW values.

## Figures and Tables

**Table 1:** Summary of the demographic and clinical characteristics of the study group.

Parameter	Study group (IQR)
Age (years)	40 ± 17.89 (range 18–93)
Sex (female/male)	14/158 (8% vs. 92%)
Creatinine (mg/dL)	0.83 (IQR: 0.69–1.04)
WBC (10^3^/mm^3^)	13.21 (IQR: 10.65–17.54)
RDW (%)	13.3 (IQR: 12.80–14.30)
Total protein (g/dL)	6.98 (IQR: 6.50–7.41)
Albumin (g/dL)	4.15 (IQR: 3.48–4.40)
Perforation diameter (mm)	5 (IQR: 5.00–10.00)
Hospital stay (days)	5 (IQR: 5.00–7.00)

Data are presented as median value (interquartile range). IQR, interquartile range.

**Table 2:** Sensitivity, specificity, positive likelihood ratio (+LR), negative likelihood ratio (LR), positive predictive value, and negative predictive value of RDW for mortality at the optimal cutoff level.

RDW	Sensitivity (95% CI)	Specificity (95% CI)	+LR	–LR	Positive predictive value (95% CI)	Negative predictive value (95% CI)
|>14.25	80.00 (51.91–95.67)	82.17 (75.27–87.81)	4.49	0.24	30.00 (21.96–39.49)	97.73 (93.97–99.16)

**Table 3:** Overview of predictive systems.

Boey, 1987	Patients with PUP	30 day mortality	Presentation within or after 24 hours; shock; level of comorbidity.

PULP, 2012	Patients with PUP	30 day mortality	Presentation within or after 24 hours; shock; ASA score, active malignancy, liver insufficiency; serum creatinine > 130 mmol/L; acquired immune deficiency syndrome

ASA, 1941	General surgical population	Preoperative risk assessment for surgical patients	Degree of comorbidity and systemic disease

## Data Availability

The data used to support the findings of this study are available from the corresponding author upon reasonable request with the permission of the institute.
